# Re-ascent triggered high-altitude pulmonary and cerebral edema in a Tibetan with pre-existing high-altitude polycythemia: a Case Report

**DOI:** 10.3389/fphys.2025.1685329

**Published:** 2026-01-15

**Authors:** He Huang, Shuaijun Yuan, Limin Zhang, Jiaye Song, Dengwei Xue, Dongmei Liu, Jingxin Cao

**Affiliations:** 1 Department of Neurology, The 940th Hospital of Joint Logistics Support Force of the Chinese People’s Liberation Army, Lanzhou, China; 2 Department of Respiratory, Affiliated Fukang Hospital of Tibet University, Lhasa, China; 3 Department of Preventive Medicine, Tibet University, Lhasa, China; 4 Department of Critical Care Medicine, The 940th Hospital of Joint Logistics Support Force of the Chinese People’s Liberation Army, Lanzhou, China

**Keywords:** high-altitude cerebral edema, high-altitude polycythemia, high-altitude pulmonary edema, re-ascent, susceptibility, Tibetan population

## Abstract

High-altitude pulmonary edema (HAPE), high-altitude cerebral edema (HACE), and high-altitude polycythemia (HAPC) are each rarely observed in Tibetan populations. The coexistence of HAPE, HACE, and HAPC in the same person has not been previously documented. Here, we report the case of a native Tibetan male with HAPC who developed both HAPE and HACE upon re-ascent to an altitude of 3,650 m after a 27-day stay at low altitude. On the 3rd-4th day post-return, the patient exhibited persistent dyspnea, chest tightness, hypersomnia, intermittent agitation, and confusion. Chest CT and multimodal neuroimaging confirmed the presence of HAPE and HACE. Treatment followed guidelines (supplemental oxygen, high-dose dexamethasone) along with supportive measures, resulting in clinical resolution. This is the first reported case of co-occurring HAPE, HACE and HAPC in a native Tibetan upon re-ascent, suggesting that pre-existing HAPC may be a significant risk factor for severe acute high-altitude illness in this setting.

## Introduction

High-altitude pulmonary edema (HAPE) and high-altitude cerebral edema (HACE) are life-threatening acute high-altitude illnesses, typically affecting non-acclimatized lowlanders ascending rapidly above 2,500 m (Gatterer et al., 2024). In contrast, high-altitude polycythemia (HAPC) is a common chronic high-altitude illness characterized by excessive erythrocytosis due to maladaptation to prolonged hypoxia, which lead to increased blood viscosity, impaired microcirculation, thrombosis, and hypoxic organ damage (Adams and Peel, 2025; Garrido et al., 2021; Zhou et al., 2025). While these disorders predominantly affect lowlanders entering high altitudes, they are exceptionally rare in genetically high-altitude adapted populations like Tibetans (Ge, 2025; Gonzales, 2025; Zhang et al., 2025).

Here, we report a native Tibetan man with HAPC who developed HAPE and HACE upon re-ascent to 3,650 m. To our knowledge, this condition has never been documented. Its occurrence in a Tibetan carries important clinical implications, suggesting HAPC patients in high-altitude regions face greater risk of severe acute high-altitude illness than previously recognized. Furthermore, this case offers valuable insights into the pathophysiological mechanisms associated with the concurrent development of HAPE and HACE in the context of pre-existing HAPC, and underscores acute illness risks during re-ascent in this population.

### Case presentation

A 36-year-old male of Tibetan ancestry was born and raised at 4,200 m in Tibet. He was a singer of traditional Tibetan opera (non-manual laborer). In 2022, he noted symptoms of high-altitude maladaptation such as exertional dyspnea, fatigue, palpitations, and cyanosis, and was diagnosed with HAPC at the local hospital. These symptoms markedly improved after relocation to Lhasa (elevation: 3,650 m) in 2022 (Supplementary Figure S1).

From February 7 to 6 March 2025, he traveled for 27 days at a low altitude (elevation: 500 m), during which there were no discomfort symptoms. On 9 March 2025, he returned to Lhasa (elevation: 3,650 m), and rested at home without physical exertion. 7 h post-return, he progressively developed headache, dizziness, nausea, vomiting, and productive cough. No medical consultation was sought. On the 3rd-4th day post-return (March 11–12, 2025), his initial symptoms worsened with new-onset persistent palpitations, dyspnea, chest tightness, hypersomnia, intermittent agitation, and confusion. Home oxygen self-administration provided partial relief, but he declined hospital evaluation. On the 5th day post-return (13 March 2025), he visited the Respiratory Department of Fukang Hospital (Lhasa, Tibet, China) due to unremitting symptoms (Supplementary Figure S1).

**FIGURE 1:**
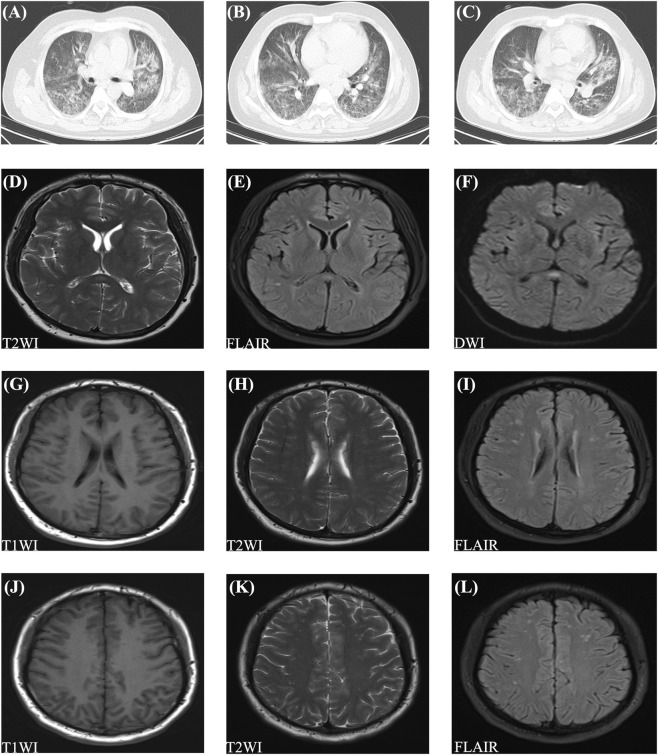
Chest CT and cranial MRI scans of the patient. **(A–C)** Chest CT showing patchy and flocculent opacities in both lungs. **(D–F)** Cranial MRI demonstrating nodular hyperintense on T2WI, FLAIR and DWI in the splenium of corpus callosum. **(G–I)** Cranial MRI showing multiple patchy hypointense on T1WI, and hyperintense on T2WI and FLAIR around periventricular. **(J–L)** Cranial MRI showing multiple patchy hypointense on T1WI, and hyperintense on T2WI and FLAIR around semioval center. CT: computed tomography; MRI: magnetic resonance imaging; T2WI: T2-weighted imaging; FLAIR: fluid attenuated inversion recovery; DWI: diffusion-weighted imaging; T1WI: T1-weighted imaging.

Physical examination revealed temperature 37.1 °C, heart rate 116 beats/min, respiratory rate 31 breaths/min, and blood pressure 136/87 mmHg; peripheral oxygen saturation (SpO_2_) at room air was critically low at 63%. The patient was alert but lethargic with delayed responsiveness, exhibiting plethoric facies and severe lip/nailbed cyanosis. Auscultation of the lungs demonstrated diminished breath sounds with diffuse bilateral crackles. Chest CT showed patchy and flocculent opacities in both lungs, consistent with HAPE (Figures 1A–C). Cranial MRI showed nodular hyperintense on T2WI, FLAIR and DWI in the splenium of corpus callosum (Figures 1D–F). It also revealed multiple patchy hypointense on T1WI, and hyperintense on T2WI and FLAIR around periventricular and semioval center (Figures 1G–L). These results of cranial MRI suggested mild HACE. The abnormal results of laboratory test demonstrated systemic inflammation, potential infection risk, HAPC, and hemolysis (detailed results shown in Table 1). Electrocardiogram showed sinus tachycardia. The remaining auxiliary examinations, including blood biochemistry, blood coagulation indicators, routine stool examination, respiratory virus nucleic acid testing, echocardiography and abdominal ultrasound were all within the normal range.

**TABLE 1:** The abnormal laboratory results of the patient on admission (13 March 2025).

Parameters	Results	Reference interval	Interpretation
Inflammatory indicators			
WBC (10^9^/L)	23.06	3.5–9.5	Inflammation
NEU (10^9^/L)	20.73	1.8–6.3	
CRP (mg/L)	31.43	0–10	
PCT (ng/mL)	0.51	≤0.05	Infection
Erythrocyte parameter			
RBC (10^12^/L)	7.23	5.3–5.8	HAPC
HGB (g/L)	243.00	130–175	
HCT (%)	74.90	40–50	
Biochemical indexes			
TBIL (μmol/L)	37.1	≤26	Hemolysis
DBIL (μmol/L)	15.2	≤8	
UBIL (μmol/L)	21.9	<18	
Urinalysis parameters			
Urobilinogen	2+	Negative	Hemolysis/inflammation
Protein	1+	Negative	
Bilirubin	1+	Negative	

WBC: white blood cell count; NEU: blood neutrophils count; CRP: C-reactive protein; PCT: procalcitonin; RBC: red blood cell count; HGB: hemoglobin; HCT: hematocrit; TBIL: total bilirubin; DBIL: direct bilirubin; UBIL: unconjugated bilirubin.

Based on the patient’s symptoms, signs and auxiliary examination results, he was diagnosed with HAPE, HACE and HAPC. The patient received guideline-based therapy consisting of continuous low-flow oxygen (two to three L/min) and high-dose intravenous dexamethasone (5 mg daily) (Keller et al., 1995; Luks et al., 2024; Maggiorini et al., 2006; Siebenmann et al., 2011). Supportive treatment included intravenous aminophylline (0.25 g twice daily) for bronchodilation and intravenous ceftazidime (2 g thrice daily) for empirical antimicrobial coverage. By the 3rd day of hospitalization (15 March 2025), the patient achieved complete neurological symptom resolution. At discharge (22 March 2025), vital signs and laboratory results normalized (Table 2) with radiographic resolution of pulmonary/cerebral edema. The 1-month follow-up confirmed no recurrence of HAPE/HACE.

**TABLE 2:** Longitudinal changes in vital signs and laboratory results of the patient.

Parameters	Results				Reference interval
	March 13 (admission)	March 15	March 18	March 22 (discharge)	
Vital signs					
Temp (°C)	37.1	36.3	36.4	36.4	36.5–37.5
RR (breaths/min)	31	21	20	19	12–20
HR (beats/min)	116	79	76	70	60–100
SBP (mmHg)	136	119	114	126	90–140
DBP (mmHg)	87	87	85	70	60–90
SpO_2_ (%)	63^*^	90^#^	92^#^	90^*^	88–92^$^
Inflammatory indicators					
WBC (10^9^/L)	23.06	11.51	8.13	5.68	3.5–9.5
NEU (10^9^/L)	20.73	10.01	6.12	4.22	1.8–6.3
CRP (mg/L)	31.43	26.2	10.93	<10.00	0–10
PCT (ng/mL)	0.51	0.21	0.17	0.09	≤0.05
Erythrocyte parameters					
RBC (10^12^/L)	7.23	6.50	7.04	6.99	5.3–5.8
HGB (g/L)	243.00	212.00	222.00	224.00	130–175
HCT (%)	74.90	66.80	73.50	72.1	40–50
Biochemical indexes					
TBIL (μmol/L)	37.1	29.3	Not tested	17.5	≤26
DBIL (μmol/L)	15.2	12.0	Not tested	6.6	≤8
UBIL (μmol/L)	21.9	17.3	Not tested	10.9	<18
Urinalysis parameters					
Urobilinogen	2+	Not tested	Not tested	-	Negative
Protein	1+	Not tested	Not tested	-	Negative
Bilirubin	1+	Not tested	Not tested	-	Negative

Temp: temperature; RR: respiratory rate; HR: heart rate; SBP: systolic blood pressure; DBP: diastolic blood pressure; SpO_2_: peripheral oxygen saturation; WBC: white blood cell count; NEU: blood neutrophils count; CRP: C-reactive protein; PCT: procalcitonin; RBC: red blood cell count; HGB: hemoglobin; HCT: hematocrit; TBIL: total bilirubin; DBIL: direct bilirubin; UBIL: unconjugated bilirubin.

*: SpO_2_ measurements were tested with room air.

#: SpO_2_ measurements were tested under continuous low-flow oxygen therapy (2–3 L/min).

$: Normal value of SpO_2_ for Tibetan residents at an altitude of 3,500 to 4,000 m (West et al., 2007).

## Discussion

This report presents the first documented case of concurrent, definitively diagnosed HAPE and HACE triggered by re-ascent in a native Tibetan with pre-existing HAPC. While severe acute high-altitude illness is exceptionally rare in adapted populations, this case underscores that such illness resistibility is not absolute. Although rare, re-entry HAPE has been reported in Tibetan individuals (Baniya et al., 2017; Wu, 2004). A prior case by Wu et al. described a Tibetan man with chronic mountain sickness who developed HAPE upon re-ascent to an altitude of 4,300 m (Wu, 2004). Our case, featuring the full triad of HAPC, HAPE, and HACE, suggests that pre-existing HAPC may represent a critical predisposing factor, significantly amplifying the risk of severe acute high-altitude illness upon re-ascent.

The pathophysiological link likely centers on the profound physiological burden imposed by HAPC. Marked erythrocytosis creates a state of severe hyperviscosity and impaired microcirculatory flow (Leon-Velarde et al., 2003; Manier et al., 1988; Zhou et al., 2025). This compromises oxygen delivery and blunts hypoxic ventilatory responses, leading to profound hypoxemia upon re-ascent. Such severe hypoxemia exacerbates hypoxic pulmonary vasoconstriction, increasing pulmonary capillary pressure and permeability—key steps in HAPE pathogenesis (Archer et al., 2024; Bärtsch et al., 2005; Dunham-Snary et al., 2017; Li et al., 2018; Maggiorini et al., 2001). Concurrently, it promotes cerebral endothelial dysfunction and blood-brain barrier disruption, contributing to HACE development (Li et al., 2018; Xue et al., 2022). Moreover, hypoxia-driven amplification of the pre-existing inflammatory state in HAPC likely exacerbates vascular leakage in both the pulmonary and cerebral circulations upon re-ascent (Song et al., 2016; Wu et al., 2018; Yi et al., 2021; Zhou et al., 2017).

This case yields four critical clinical insights: (I) Even high-altitude adapted Tibetan populations are not protected from HAPE and HACE upon re-ascent; (II) Pre-existing HAPC likely represents a novel risk factor for HAPE/HACE; (III) Emergent chest CT and multimodal neuroimaging are essential for the rapid and accurate diagnosis of concurrent HAPE-HAC; and (IV) Guideline-based therapy consisting of continuous low-flow oxygen and high-dose dexamethasone effectively controls disease progression (Luks et al., 2024). Furthermore, we postulate that HAPC patients’ risk of developing HAPE/HACE upon re-ascent may exceed current recognition, warranting future large-scale studies to precisely quantify risks in HAPC patients after low-altitude sojourns and develop targeted preventive strategies.

This study is limited by the lack of longitudinal cardiopulmonary data. Consequently, follow-up assessments incorporating echocardiography to evaluate pulmonary artery pressure and right heart adaptation are necessary. Moreover, the extreme hematologic findings justify genetic investigation to elucidate the basis of the patient’s HAPC, including screening for variants associated with conditions like Chuvash polycythemia (Gangat et al., 2021).

In conclusion, this first reported case of HAPC, HAPE, and HACE co-occurrence in a Tibetan native upon re-ascent, challenges the conventional view of severe acute high-altitude illness resistibility. Furthermore, our findings indicate significant risks for HAPC patients upon re-ascent to high altitude.

## Data Availability

The raw data supporting the conclusions of this article will be made available by the authors, without undue reservation.
