# Supplementary material for: Re-ascent triggered high-altitude pulmonary and cerebral edema in a Tibetan with pre-existing high-altitude polycythemia: a Case Report
Source: Front Physiol. 2026 Jan 15;16:1685329. doi: 10.3389/fphys.2025.1685329 (PMC12852030; doi:10.3389/fphys.2025.1685329)
Supplement: Supplementary file 1 [file Table1.docx]

***Supplementary Material***

**Supplementary Figure 1.** A timeline of disease progression for the patient.

HAPC: high-altitude polycythemia; HAPE: high-altitude pulmonary edema; HACE: high-altitude cerebral edema.
