# Supplementary figures and images for: Re-ascent triggered high-altitude pulmonary and cerebral edema in a Tibetan with pre-existing high-altitude polycythemia: a Case Report
Source: Front Physiol. 2026 Jan 15;16:1685329. doi: 10.3389/fphys.2025.1685329 (PMC12852030; doi:10.3389/fphys.2025.1685329)

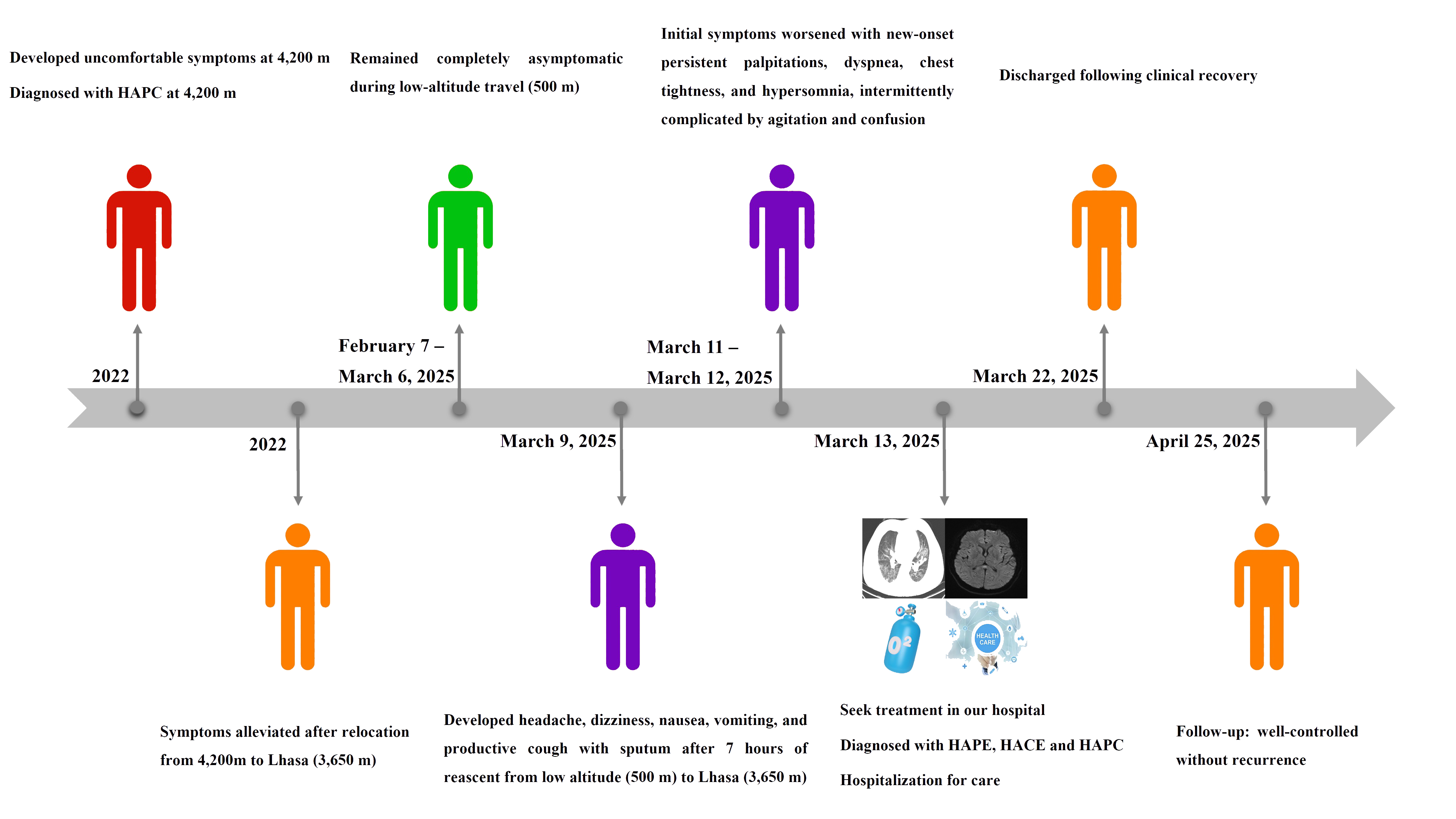

Supplement: Supplementary file 2 [file Image1.tif]
